# The Duffy-null genotype and risk of infection

**DOI:** 10.1093/hmg/ddaa208

**Published:** 2020-09-22

**Authors:** Sophie E Legge, Rune H Christensen, Liselotte Petersen, Antonio F Pardiñas, Matthew Bracher-Smith, Steven Knapper, Jonas Bybjerg-Grauholm, Marie Baekvad-Hansen, David M Hougaard, Thomas Werge, Merete Nordentoft, Preben Bo Mortensen, Michael J Owen, Michael C O’Donovan, Michael E Benros, James T R Walters

**Affiliations:** MRC Centre for Neuropsychiatric Genetics and Genomics, Division of Psychological Medicine and Clinical Neurosciences, School of Medicine, Cardiff University, Cardiff CF24 4HQ, UK; Copenhagen Research Centre for Mental Health, Mental Health Centre Copenhagen, Copenhagen University Hospital, Copenhagen 2605, Denmark; National Centre for Register-Based Research, Department of Economics and Business Economics, Aarhus University, Aarhus, 8210, Denmark; Centre for Integrated Register-Based Research, Aarhus University, Aarhus, 8210, Denmark; The Lundbeck Foundation for Integrative Psychiatric Research (iPSYCH), Aarhus University, Aarhus 8210, Denmark; MRC Centre for Neuropsychiatric Genetics and Genomics, Division of Psychological Medicine and Clinical Neurosciences, School of Medicine, Cardiff University, Cardiff CF24 4HQ, UK; MRC Centre for Neuropsychiatric Genetics and Genomics, Division of Psychological Medicine and Clinical Neurosciences, School of Medicine, Cardiff University, Cardiff CF24 4HQ, UK; Division of Cancer and Genetics, School of Medicine, Cardiff University, Cardiff CF14 4XN, UK; Centre for Neonatal Screening, Department for Congenital Disorders, Statens Serum Institut, Copenhagen 2300, Denmark; Centre for Neonatal Screening, Department for Congenital Disorders, Statens Serum Institut, Copenhagen 2300, Denmark; Centre for Neonatal Screening, Department for Congenital Disorders, Statens Serum Institut, Copenhagen 2300, Denmark; Institute of Biological Psychiatry, Mental Health Centre Sct. Hans, Mental Health Services Copenhagen, Roskilde 4000, Denmark; Copenhagen Research Centre for Mental Health, Mental Health Centre Copenhagen, Copenhagen University Hospital, Copenhagen 2605, Denmark; The Lundbeck Foundation for Integrative Psychiatric Research (iPSYCH), Aarhus University, Aarhus 8210, Denmark; National Centre for Register-Based Research, Department of Economics and Business Economics, Aarhus University, Aarhus, 8210, Denmark; Centre for Integrated Register-Based Research, Aarhus University, Aarhus, 8210, Denmark; The Lundbeck Foundation for Integrative Psychiatric Research (iPSYCH), Aarhus University, Aarhus 8210, Denmark; MRC Centre for Neuropsychiatric Genetics and Genomics, Division of Psychological Medicine and Clinical Neurosciences, School of Medicine, Cardiff University, Cardiff CF24 4HQ, UK; MRC Centre for Neuropsychiatric Genetics and Genomics, Division of Psychological Medicine and Clinical Neurosciences, School of Medicine, Cardiff University, Cardiff CF24 4HQ, UK; Copenhagen Research Centre for Mental Health, Mental Health Centre Copenhagen, Copenhagen University Hospital, Copenhagen 2605, Denmark; Department of Immunology and Microbiology, Faculty of Health and Medical Sciences, University of Copenhagen, Copenhagen 2200, Denmark; MRC Centre for Neuropsychiatric Genetics and Genomics, Division of Psychological Medicine and Clinical Neurosciences, School of Medicine, Cardiff University, Cardiff CF24 4HQ, UK

## Abstract

Many medical treatments, from oncology to psychiatry, can lower white blood cell counts and thus access to these treatments can be restricted to individuals with normal levels of white blood cells, principally in order to minimize risk of serious infection. This adversely affects individuals of African or Middle Eastern ancestries who have on average a reduced number of circulating white blood cells, because of the Duffy-null (CC) genotype at rs2814778 in the *ACKR1* gene. Here, we investigate whether the Duffy-null genotype is associated with the risk of infection using the UK Biobank sample and the iPSYCH Danish case-cohort study, two population-based samples from different countries and age ranges. We found that a high proportion of those with the Duffy-null genotype (21%) had a neutrophil count below the threshold often used as a cut-off for access to relevant treatments, compared with 1% of those with the TC/TT genotype. In addition we found that despite its strong association with lower average neutrophil counts, the Duffy-null genotype was not associated with an increased risk of infection, viral or bacterial. These results have widespread implications for the clinical treatment of individuals of African ancestry and indicate that neutrophil thresholds to access treatments could be lowered in individuals with the Duffy-null genotype without an increased risk of infection.

## Introduction

It has long been recognized that individuals with an African or certain Middle Eastern ancestries often have reduced numbers of white blood cells, specifically neutrophils, compared with those with European ancestries (1). When neutrophil counts are <1.5 × 10^9^/L and the individual has no serious or recurrent infections, this condition is termed benign ethnic neutropenia. The Duffy-null (CC) genotype at rs2814778, in the Atypical Chemokine Receptor 1 (*ACKR1*) gene, previously known as *FY* and *DARC*, has been robustly associated with reduced neutrophil counts in individuals of African ancestry (2,3) and is considered to be the cause of benign ethnic neutropenia (4). The Duffy-null genotype confers an evolutionary advantage by protecting against the malaria parasite *Plasmodium vivax* infection (5), and thus it is highly prevalent in geographical areas previously endemic for malaria, such as sub-Saharan Africa. The Duffy-null genotype has a prevalence of ~80% in Black African/Caribbean populations in the UK (6), ~65% prevalence in African Americans (3), and is very rare in individuals of European ancestry.

Recent studies have indicated that the Duffy-null genotype causes an altered neutrophil morphology that leads to neutrophils egressing from circulating blood into tissues and thus causing neutropenia (7,8). This mechanism is thought to be clinically benign because the production and functioning of neutrophils is not reduced and so their ability to fight infection remains unchanged (4). It has therefore been assumed that the Duffy-null genotype does not lead to increased rates of infection despite its association with reduced neutrophil counts. However, there have only been a few studies assessing infection outcomes in small clinical cohorts (<50 participants) of clinically diagnosed individuals with benign ethnic neutropenia (9–11), and none to date for the Duffy-null genotype (12).

Disparities in access to health care and treatment outcomes, including mortality, between individuals of African and European ancestries living in Europe and the United States are well documented (13) and unrecognized benign neutropenia could add substantially to this disparity. For example, individuals of African ancestry have been shown to have poorer clinical outcomes and access to medications for which neutropenia is a barrier (14,15). Thus, establishing that the neutropenia because of the Duffy-null genotype is benign has important clinical implications across the world for many treatments such as chemotherapy, immunosuppressant therapy, organ transplantation and antipsychotics such as clozapine (16). To address this knowledge gap, the aim of this study is to establish whether individuals with the Duffy-null genotype have increased rates of infection in two population-based samples from different countries and age ranges; the UK Biobank (17) and the iPSYCH Danish case-cohort study (18).

## Results

In the UK Biobank sample, 7450 (1.53%) individuals had the CC (Duffy-null) genotype at rs2814778, 4525 (0.93%) had the TC genotype and 475 348 (97.54%) had the TT genotype. As expected, the great majority of individuals with the CC genotype reported their ethnicity as Black African/Caribbean (86.0%, Supplementary Material, Table S1). A total of 7644 individuals with a self-reported Black African/Caribbean ethnicity were selected for our primary analysis (57.05% female, mean age of 51.91 years at recruitment), of which 6363 (83.24%) had the CC genotype. Analysis of genetic principal components supports the finding that the CC genotype is not completely congruent with self-reported Black African/Caribbean ethnicity or our definition of African ancestry (Supplementary Material, Fig. S1). Supplementary Material, Table S2 details the country of birth for individuals with the CC genotype in UK Biobank.

A total of 283/77880 (0.36%) individuals in the iPSYCH sample had the CC (Duffy-null) genotype. Of these, 228 (80.57%) had 2 parents of African origin, 27 (9.54%) had 2 parents of Middle Eastern origin and 28 (9.89%) had parents of mixed or another origin. We selected 281 individuals for inclusion in the study that had 2 parents of African ancestry specifically from countries at risk for malaria (42.45% female), of whom 217 (77.2%) had the CC genotype and 64 (22.8%) the TC/TT genotype for rs2814778 (Supplementary Material, Table S3). The majority of study individuals was born between 1995 and 2005 and were followed for an average of 7.4 years (Supplementary Material, Fig. S2 and Table S4).

### Duffy-null genotype and neutrophil counts

**Figure 1:**
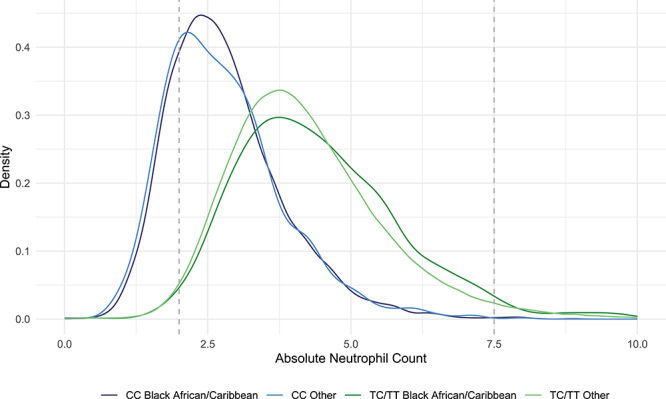
Neutrophil count in individuals with the CC (Duffy-null) and TC/TT genotypes at rs2814778. Density plot of absolute neutrophil count (ANC) in individuals with the CC (Duffy-null) and TC/TT genotypes at rs2814778 and self-reported ethnicity in UK Biobank participants. Other category includes all self-reported ethnicities other than Black African/Caribbean. The dotted grey lines represent the current UK thresholds for the normal adult range of ANC (2.0–7.5 × 10^9^/L).

Figure 1 displays the distribution of absolute neutrophil count (ANC) for individuals with the CC (Duffy-null) and TC/TT genotype at rs2814778 in UK Biobank. The CC genotype was strongly associated with lower ANC in individuals of Black African/Caribbean ethnicity (β = −1.61; 95% confidence interval (CI) = −1.70, −1.53; *P* = 3.52 × 10^−293^) and also in individuals of other ethnicities (β = −1.72; 95% CI = −1.86, −1.58; *P* = 4.41 × 10^−129^). For individuals reporting a Black African/Caribbean ethnicity, the mean ANC for individuals with the CC and TC/TT genotype was 2.82 × 10^9^/L (standard deviation (SD) = 1.02) and 4.43 × 10^9^/L (SD = 1.41), respectively. Table 1A provides comparative proportions of individuals with the CC and TC/TT genotypes that fall below certain ANC thresholds. Individuals with the CC genotype were significantly more likely to have an ANC between 0 and 2.0 × 10^9^/L, the current UK threshold for normal adult range of ANC, (odds ratio (OR) = 25.46; 95% CI = 14.39, 45.06; *P* = 1.06 × 10^−28^) and between 0 and 1.5 × 10^9^/L (OR = 45.88; 95% CI = 12.09, 174.07; *P* = 1.86 × 10^−8^) (Table 1B). The results remained consistent in analyses including all ethnicities (Supplementary Material, Results).

**Table 1:** Duffy-null genotype and absolute neutrophil count

**A**	CC genotype		TC/TT genotype	
	Black African/Caribbean	Other ethnicity	Black African/Caribbean	Other ethnicity
Total N with ANC data	6002	964	1220	458 077
Mean ANC [SD]	2.82 [1.02]	2.79 [1.05]	4.43 [1.41]	4.22 [1.34]
ANC > 7.5	13 (0.22%)	1 (0.10%)	32 (2.62%)	10 774 (2.35%)
ANC 2.0–7.5	4751 (79.16%)	735 (76.24%)	1173 (96.15%)	440 012 (96.06%)
ANC 1.5–2.0	935 (15.58%)	172 (17.84%)	12 (0.98%)	5599 (1.22%)
ANC 1.0–1.5	272 (4.53%)	48 (4.98%)	1 (0.08%)	981 (0.21%)
ANC 0.5–1.0	28 (0.47%)	7 (0.73%)	1 (0.08%)	380 (0.08%)
ANC < 0.5	3 (0.05%)	1 (0.10%)	1 (0.08%)	331 (0.07%)
**B**				
**Black African/Caribbean ethnicity**				
	**CC (*n* = 6002)**	**TC/TT (*n* = 1220)**	**Odds ratio (95% CI)**	***P***
ANC < 2.0	1238 (20.63%)	15 (1.23%)	25.46 (14.39–45.06)	1.06 × 10^−28^
ANC < 1.5	303 (5.05%)	3 (0.25%)	45.88 (12.09–174.07)	1.86 × 10^−8^
**All ethnicities combined**				
	**CC (*n* = 6966)**	**TC/TT (*n* = 459 297)**	**Odds ratio (95% CI)**	***P***
ANC < 2.0	1466 (21.05%)	7306 (1.59%)	32.44 (22.86–46.04)	1.37 × 10^−84^
ANC < 1.5	359 (5.15%)	1695 (0.37%)	45.22 (23.31–87.73)	1.78 × 10^−29^

Association between the Duffy-null genotype and absolute neutrophil count (ANC) in UK Biobank participants. **A** provides comparative proportions of individuals with the CC and TC/TT genotypes that fall below certain ANC thresholds. Columns represent ANC thresholds, values for individuals with the CC (Duffy-null) and TT/TC genotypes, respectively, for rs2814778 with (i) a self-reported Black African/Caribbean ethnicity, and (ii) self-reported ‘other’ (non-Black African/Caribbean) ethnicity. **B** details the association between the CC (Duffy-null) genotype and an ANC < 2.0 × 10^9^/L and 1.5 × 10^9^/L. CI, confidence interval.

### Duffy-null genotype and risk of infection

Rates of infection for individuals with the CC (Duffy-null) and TC/TT genotype for rs2814778 in the UK Biobank and iPSYCH samples are detailed in Table 2. In UK Biobank, the CC genotype did not increase the risk of infection in individuals with a Black African/Caribbean ethnicity (rate ratio (RR) = 0.96; 95% CI = 0.82, 1.13; *P* = 0.61) or the number of infections per subject (RR = 0.95; 95% CI = 0.83, 1.08; *P* = 0.42). In the iPSYCH cohort, individuals with the CC genotype did not have an increased risk of infection (RR = 0.97; 95% CI = 0.65, 1.45; *P* = 0.88).

**Table 2:** Duffy-null genotype and risk of infection

Any infection: UK Biobank				
	**CC (*n* = 6345)**	**TC/TT (*n* = 1281)**	**Rate ratio (95% CI)**	***P***
Any infection	1188 (18.72%)	285 (22.23%)	0.96 (0.82–1.13)	0.61
Number of infections per subject	0.27	0.30	0.95 (0.83–1.08)	0.42
**Any infection: iPSYCH**				
	**CC (*n* = 217)**	**TC/TT (*n* = 64)**	**Rate ratio (95% CI)**	***P***
Any infection	112 (51.6%)	33 (51.6%)	0.97 (0.65–1.45)	0.88
Av. observation years per subject	7.2	7.5		
**Type of infection: UK Biobank**				
	**CC (*n* = 6345)**	**TC/TT (*n* = 1281)**	**Rate ratio (95% CI)**	***P***
Bacterial infection	593 (9.35%)	139 (10.85%)	0.94 (0.75–1.18)	0.61
Viral infection	275 (4.33%)	59 (4.61%)	1.03 (0.73–1.45)	0.88
Other infection	366 (5.77%)	86 (6.71%)	0.89 (0.67–1.20)	0.46
**Site of infection: UK Biobank**				
	**CC (*n* = 6345)**	**TC/TT (*n* = 1281)**	**Rate ratio (95% CI)**	***P***
Respiratory	384 (6.05%)	88 (6.87%)	0.96 (0.72–1.28)	0.79
Skin	235 (3.70%)	60 (4.68%)	0.85 (0.60–1.21)	0.38
Gastrointestinal	208 (3.28%)	50 (3.90%)	0.98 (0.67–1.43)	0.91
Sepsis	66 (1.04%)	13 (1.01%)	1.11 (0.54–2.27)	0.78
Hepatitis	58 (1.34%)	13 (1.01%)	1.00 (0.48–2.07)	0.99

Association between Duffy-null genotyoe and risk of infection in individuals with Black African/Caribbean ethnicity in (i) the UK Biobank sample and (ii) the iPSYCH sample. Only sites of infection that were present in at least 1% of controls were included. Details of analysis conducted in whole UK Biobank sample (all ethnicities) are given in Supplementary Material, Table S5.

**Figure 2:**
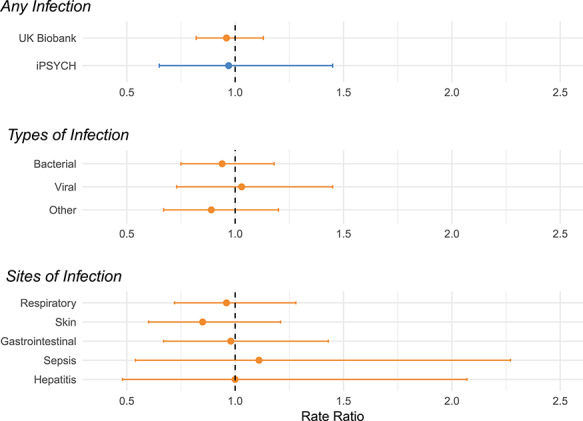
Association between risk of infection and the Duffy-null genotype. The association between risk of infection and the Duffy-null genotype. The x-axis displays the rate ratio; values < 1 indicate a reduced risk of infection and values > 1 indicate an increased risk. ICD codes used to define infection categories are listed in Supplementary Material, Table S9. ‘Other’ infections include infections not specified as bacterial or viral such as protozoa infections and those caused by contaminated food and water.

In the UK Biobank, we similarly found no association between genotype and specific types (bacterial, viral) or anatomical sites (respiratory, skin, gastrointestinal) of infection (Fig. 2, Table 2). Furthermore, individuals with the CC genotype in comparison to the TC/TT genotype in UK Biobank were not significantly more likely to have died from an infection-related illness (0.20% (*n* = 13) vs. 0.16% (*n* = 2); RR = 1.30; 95% CI = 0.33, 5.18; *P* = 0.71). All UK Biobank results remained consistent in analyses including all ethnicities (Supplementary Material, Results and Table S5).

All UK Biobank analyses controlled for sex, age at recruitment, Townsend deprivation index at recruitment and the first 20 genetic principal components to control for population structure. Although these covariates were not associated with increased rates in infections when analyses were restricted to individuals with a Black African/Caribbean ethnicity, when individuals of all ethnicities were included in analyses, the Townsend deprivation index was strongly associated with an increased risk of infection (RR = 1.12; 95% CI = 1.11, 1.12; *P* = 2.56 × 10^−262^), as was older age (RR = 1.12; 95% CI = 1.11, 1.13; *P* = 2.13 × 10^−253^). A Black African/Caribbean ethnicity was not associated with increased rates of infection (RR = 0.96; 95% CI = 0.92, 1.02; *P* = 0.16).

### Ranges of neutrophil counts and risk of infection

**Figure 3:**
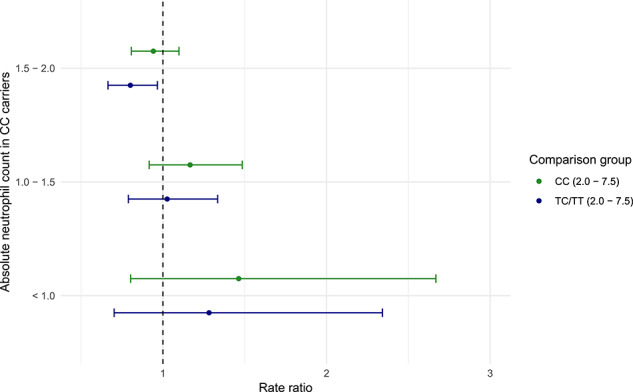
Association between risk of infection and the Duffy-null genotype at low neutrophil counts. Risk of infection for individuals with the CC (Duffy-null) genotype at rs2814778 who had neutrophil counts below the currently accepted range (2.0–7.5 × 10^9^/L) in contrast to (i) individuals with the CC genotype and a neutrophil count within the currently accepted range and (ii) individuals with the TC/TT genotype who had a neutrophil count within the currently accepted range. The x-axis displays the rate ratio; values < 1 indicate a reduced risk of infection and values > 1 indicate an increased risk.

Lastly, we investigated the risk of infection for different ranges of ANC in individuals of all ethnicities in UK Biobank. Figure 3 and Table 3 details the risk of infection in individuals with the CC genotype and a low ANC in contrast to individuals with the CC and TC/TT genotype with a normal ANC (2.0–7.5 × 10^9^/L). Individuals with the CC genotype and an ANC between 1.5 and 2.0 × 10^9^/L did not have an increased risk of infection in contrast to individuals with a normal ANC who had the CC genotype (17.4% vs. 18.8%; RR = 0.94; 95% CI = 0.81, 1.10; *P* = 0.44), and had a lower risk of infection compared with the TC/TT genotype (17.4% vs. 19.6%; RR = 0.80; 95% CI = 0.66, 0.97; *P* = 0.02). However, individuals with the TC/TT genotype and an ANC between 1.5 and 2.0 × 10^9^/L did have an increased risk of infection compared with TC/TT individuals who had a normal ANC (24.1% vs. 19.6%; RR = 1.25; 95% CI = 1.10, 1.42; *P* = 5.72 × 10^−4^).

We were not able to detect any statistical difference between the rates of infection in individuals with the CC genotype and an ANC between 1.0 and 1.5 × 10^9^/L (22.0% (71/323)) compared with individuals with a normal ANC who had the CC genotype (RR = 1.17; 95% CI = 0.92, 1.48; *P* = 0.21) or the TC/TT genotype (RR = 1.03; 95% CI = 0.79, 1.33; *P* = 0.85). However the low number of subjects with an ANC <1.5 × 10^9^/L precludes us from making firm conclusions about risk of infection at these very low ANC counts (Table 3).

Supplementary Material, Table S6 details the associations between viral infections and different ranges of ANC. We found evidence indicating that individuals with low ANC have an increased risk of viral infection but that this did not differ by Duffy-null genotype. In addition, we were not able to detect any statistical difference in the risk of infection between individuals with the CC and TC/TT genotypes that had similar ANC, for example between 1.0 and 1.5 × 10^9^/L (Supplementary Material, Table S7).

## Discussion

In two samples covering different countries and age ranges, we found that the CC (Duffy-null) genotype at rs2814778, which causes a reduction in the number of circulating neutrophils, does not increase the risk of serious infection. Further studies are required to establish the risk of infection at very low ANCs, but these findings could have widespread implications for the clinical management of individuals of African ancestry.

### Main findings

Using the large UK Biobank sample, we confirmed that the Duffy-null genotype is strongly associated with reduced ANC in individuals with a self-reported Black African/Caribbean ethnicity. This has already been documented in several large meta-analyses (2,3), and we extend these findings to show that these results are consistent in individuals of other ethnicities.

Despite this large effect on ANC, we found no evidence from two independent samples that individuals with the Duffy-null genotype had an increased risk of infection. This was true for both viral and bacterial infections. Furthermore, we found no evidence of an increased risk of death from infection in those with the Duffy-null genotype. These findings provide an empirical base for the widespread assumption that the on average reduced ANC in those with the Duffy-null genotype is a benign phenomenon. Recent experimental work indicates that the lower ANC seen in individuals with the Duffy-null genotype is caused not by a reduced production of neutrophils but rather by the neutrophils egressing from circulating blood into tissues (4,7,8).

It has also been assumed that particularly low ANC (<2.0 × 10^9^/L) in those with the Duffy-null genotype is benign in nature, but there has been little evidence to confirm this assumption (9,16,19). We found that the Duffy-null genotype was not associated with an increased risk of infection for individuals with an ANC between 1.5 and 2.0 × 10^9^/L, whereas the TC/TT genotype did show an increase in infection risk. However, the low number of subjects with an ANC < 1.5 × 10^9^/L precludes us from making firm conclusions about risk of infection at these lower ANC levels, although the findings presented do not provide evidence of significantly increased rates of infections. We found that individuals with low ANC appeared to have an increased risk of viral infection, but this did not differ by Duffy-null genotype, and given that viral infections can lower ANC, this finding could be because of reverse causality if the infection was current. This analysis is further limited by the use of a single ANC measurement collected at the time of study recruitment. To conclusively address this important clinical question and to provide safe ANC thresholds that could be used in clinical settings, a prospective design is required to determine infection risk at the time of the low neutrophil count.

**Table 3:** Risk of infection in Duffy-null carriers with low neutrophil counts

Low ANC group				Comparative group (ANC 2.0–7.5 × 10^9^/L)					
Genotype	ANC range (10^9^/L)	Number of subjects (*N*)	Number with infections (*N*%)	Genotype	Number of subjects (*N*)	Number with infections (*N*%)	RR (95% CI)	SE	*P*
CC	<1.0	39	11 (28.21%)	CC	5523	1039 (18.81%)	1.46 (0.80–2.67)	0.31	0.214
				TC/TT	444 715	87 295 (19.63%)	1.28 (0.70–2.34)	0.31	0.419
CC	1.0–1.5	323	71 (21.98%)	CC	5523	1039 (18.81%)	1.17 (0.92–1.48)	0.12	0.212
				TC/TT	444 715	87 295 (19.63%)	1.03 (0.79–1.33)	0.13	0.848
CC	1.5–2.0	1115	194 (17.40%)	CC	5523	1039 (18.81%)	0.94 (0.81–1.10)	0.08	0.442
				TC/TT	444 715	87 295 (19.63%)	0.80 (0.66–0.97)	0.10	0.021
TC/TT	<1.0	718	169 (23.54%)	TC/TT	444 715	87 295 (19.63%)	1.21 (1.04–1.41)	0.08	0.012
TC/TT	1.0–1.5	990	239 (24.14%)	TC/TT	444 715	87 295 (19.63%)	1.25 (1.10–1.42)	0.06	5.72 × 10^−4^
TC/TT	1.5–2.0	5659	1064 (18.80%)	TC/TT	444 715	87 295 (19.63%)	0.98 (0.93–1.05)	0.03	0.618

Association of infection in individuals with the CC genotype and a low ANC in contrast to individuals with the CC and TC/TT genotype with a normal ANC (2.0–7.5 × 10^9^/L). Columns represent firstly for low ANC groups: genotype at rs2814778, ANC range, number of subjects in said group and number of infections. The comparative group consisted of individuals with an ANC in the range of 2.0–7.5 × 10^9^/L and columns represent: genotype at rs2814778, number of subjects and number of infections. RR, rate ratio, in reference to low ANC group; SE, standard error; *P* = association *P*-value.

### Clinical implications

The findings from this study could have widespread implications for the clinical treatment of individuals with an African ancestry living in Europe and the United States, whose neutrophil count should be interpreted in the light of genotype at rs2814778. The current threshold for a ‘normal’ neutrophil count in the UK and United States is based on available normative data rather than an association with pathology or adverse effects, and is based on patients with a European ancestry. In this study, we found that 21% of individuals with the Duffy-null genotype had a neutrophil count below the currently accepted range (<2.0 × 10^9^/L) at any one time in contrast to 1.1% of those with the TC/TT genotype. Thus, these thresholds will not be appropriate for individuals with the Duffy-null genotype. Neutropenia is a contra-indication to many treatments, for example certain types of chemotherapy, immunosuppressant therapy, organ transplantation and treatment with the antipsychotic drug clozapine (16). Our findings suggest that it is likely that many individuals with an African ancestry seeking treatment in countries where they are a minority could be denied access to treatment because of a benign neutropenia caused by the Duffy-null genotype. This has important consequences; previous research shows that individuals of African ancestry living in Europe and the United States have poorer clinical outcomes and access to medications for which neutropenia is a barrier such as chemotherapy (14,15,20). Our findings suggest that ANC thresholds could be lowered to 1.5 × 10^9^/L in individuals with the Duffy-null genotype without an increased risk of infection.

For example, clozapine treatment in the UK requires a baseline ANC > 2.0 × 10^9^/L and a recent study of clozapine users found that across longitudinal neutrophil count measures, 55.4% of patients with the Duffy-null genotype had an ANC <2.0 × 10^9^/L at some point during their treatment (6). Clozapine requires regular monitoring of ANC to aid detection of a rare but potentially serious side effect of agranulocytosis. In the UK and United States, alternative monitoring thresholds for individuals with benign ethnic neutropenia have been incorporated into treatment pathways, allowing more individuals of African ancestry to access the treatment (19,21). If a prospective study could prove that individuals with the Duffy-null genotype and a low ANC did not have increased risk of infection, this strategy could be replaced with Duffy-null genotyping and extended to other treatments to aid interpretation of neutropenia in individuals of African ancestry. These adjustments could improve the racial disparities seen in healthcare outcomes.

Our findings indicate that the Duffy-null genotype, a marker of African ancestry, or self-reporting a Black African/Caribbean ethnicity, is not associated with increased rates of infection whereas in the UK Biobank sample, we observed a strong association of both age and social deprivation with increased rates of infections. We would stress that our study does not include analysis incorporating coronavirus disease-2019 infections and that these findings cannot be assumed to apply to infections other than those included in the analysis.

### Strengths and limitations

A key strength of this study is the inclusion of two independent studies with distinct characteristics that in combination give a high level of confidence in our findings. The UK Biobank sample allows for large numbers of individuals to be studied and has a wide range of data available. However, there is evidence of ascertainment bias in UK Biobank and thus the sample cannot be considered representative of the general population (22). Furthermore, given the age of UK Biobank participants was between 40 and 65 years old at the time of recruitment, sample representativeness could be limited by survivor bias; it is possible that Duffy-null carriers that were at high risk of serious infection could have suffered from disproportionate mortality. However, if this were the case we would observe lower than expected rates of individuals with the Duffy-null genotype, which we did not find. The iPSYCH cohort does not suffer from the limitations of UK Biobank, since it is a population-based birth cohort and is thus representative of the population in Denmark. However, the proportion of the iPSYCH sample with the Duffy-null genotype was low and thus more fine-grained analyses, such as specific types and sites of infection could not be conducted.

Our findings are limited to populations of African ancestry living in Europe and thus may not be generalizable to the Duffy-null carriers living in other countries. It is thus important to conduct studies of this nature in other populations. Another limitation is that our findings relate to serious infections only and it is possible that those with the Duffy-null genotype could have more frequent minor infections that do not require contact with hospitals or secondary care services.

### Conclusions

In two samples from different countries and age ranges, we found that the Duffy-null genotype, which causes an on average reduction in ANC, does not increase the risk of serious infection. This could have widespread implications for the clinical management of individuals of an African ancestry, whose neutrophil count should be interpreted in the light of genotype at rs2814778.

## Materials and Methods

### Samples

Study individuals were from two samples covering different age ranges; the UK Biobank (17) and the iPSYCH Danish case-cohort study (18). The UK Biobank is a large prospective population-based cohort study of approximately 500 000 individuals aged between 40 and 69 who were recruited from across the UK between 2006 and 2010 (17). The North West Multi-Centre Ethics Committee granted ethical approval to UK Biobank and this study was conducted under project number 13310. Primary analyses included individuals who self-reported a Black African or Black Caribbean ethnicity (UK Biobank field ID: 21000). All ancestries were included in secondary analyses.

The iPSYCH study is a population-based sample of 78 000 individuals born between 1981 and 2005 in Denmark and has been previously described (18). Ethical approval for this study was provided by the Danish Scientific Ethics Committee, the Danish Health Data Authority, the Danish data protection agency and the Danish Neonatal Screening Biobank Steering Committee. We selected individuals for this study who had two parents of African origin, specifically from countries at current or historical risk of malaria (listed in Supplementary Material, Table S8), as defined by the Danish Civil Registration System (23).

### Duffy-null genotype

UK Biobank participants were genotyped on either the UK Biobank Axiom, or the UK BiLEVE Axiom arrays at the Affymetrix Research Services Laboratory. Genotypes for rs2814778 were imputed using the Haplotype Reference Consortium panel (24) after standard quality control procedures. All genetic data were provided by UK Biobank and the imputation and quality control procedures are fully described elsewhere (25).

For the iPSYCH sample, DNA was extracted from neonatal dried blood spot samples obtained from the Danish Neonatal Screening Biobank and genotyped using the Illumina PsychChip. Genotypes for rs2814778 were imputed in 10 batches using IMPUTE2 (26) and haplotypes from the 1000 Genomes Project, phase 3 (27).

For both UK Biobank and iPSYCH samples, we selected individuals whose genotype for rs2814778 had been imputed with high confidence (genotype probability thresholds of 0–0.1, 0.9–1.1 and 1.9–2). Given the T allele for rs2814778 is dominant with respect to neutrophil count (2), individuals with the CT and TT genotypes were combined in all analyses.

### Outcome measures

#### Neutrophil count

A total of 478 511 (95.22%) UK Biobank participants had a single absolute neutrophil count (ANC) assay result (UK Biobank field ID: 30140) derived from the blood sample obtained at the initial UK Biobank assessment centre visit at which participants were recruited (between 2006 and 2010).

#### Infections

We chose as the primary study outcome a broad definition of infection, defined as any inpatient hospital admission in which at least one International Statistical Classification of Diseases and Related Health Problems (ICD) infection code was recorded (Supplementary Material, Table S9 provides the full list of codes included). Any diagnosis that started with a parent ICD code was included (for example, searching for A40 would also include A40.0, A40.1, A40.2 and so on). This included secondary infections that may not have been the primary reason for hospital contact.

Infections in the UK Biobank sample were extracted using ICD-10 codes from linked National Health Service Hospital Episode Statistics data (UK Biobank field IDs: 41270, 41 202, 41 204), linked death records from National Death Registries (UK Biobank field IDs: 40001 and 40 002), and self-report at study recruitment (UK Biobank field ID: 20002). Hospital records in UK Biobank covered inpatient admissions from 1997 until March 2017. Death records cover the period from participant recruitment to February 2018. Specific types and anatomical sites of infection were also extracted (Supplementary Material, Table S9). Death from an infection-related illness in UK Biobank was defined as an ICD infection code listed as either the primary or secondary cause of death. Number of infections was defined as the number of hospital contacts associated with any of the ICD-10 codes.

Data for infections in the iPSYCH sample were extracted from the Danish National Patient Registry (28), using ICD-8 and ICD-10 codes listed in Supplementary Material, Table S9.

### Analysis

All UK Biobank analyses were conducted controlling for sex (UK Biobank field ID: 22001), age at recruitment (UK Biobank field ID: 21022), Townsend deprivation index at recruitment (UK Biobank field ID: 189) and the first 20 genetic principal components to control for population structure. We compared the distribution of ANC between individuals with the CC (Duffy-null) and TC/TT genotypes for rs2814778 in UK Biobank via linear regression (UK Biobank field ID: 22009). Using logistic regression, we also tested the relationship between genotype and an ANC < 2.0 × 10^9^/L and 1.5 × 10^9^/L. Risk of infections were analysed by modelling (i) the occurrence of an infection and (ii) number of infections per subject using Poisson regression and adjusting for the covariates listed previously. The models were unadjusted for observation time since hospital records started in 1997 for all subjects. Our primary analyses included individuals with a self-reported Black African or Black Caribbean ethnicity but all analyses were repeated in individuals of all ethnicities to assess generalizability of the findings.

In the iPSYCH sample, we calculated rate ratios for infections in individuals with the CC and TC/TT genotype in a Cox model using robust standard errors, and adjusting for sex and psychiatric case status on the time to first infection. Subjects were followed until death, loss to follow-up or April 4, 2017 whichever occurred first. Most infections were observed before age 5.

## Supplementary Material

HMG_Duffy-null_and_Infection_Supplementary_Materials_REVISED_ddaa208

## References

[1] Haddy, T.B., Rana, S.R. and Castro, O. (1999) Benign ethnic neutropenia: what is a normal absolute neutrophil count? J. Lab. Clin. Med., 133, 15–22. 10385477 10.1053/lc.1999.v133.a94931

[2] Reich, D., Nalls, M.A., Kao, W.H., Akylbekova, E.L., Tandon, A., Patterson, N., Mullikin, J., Hsueh, W.C., Cheng, C.Y., Coresh, J. et al. (2009) Reduced neutrophil count in people of African descent is due to a regulatory variant in the Duffy antigen receptor for chemokines gene. PLoS Genet., 5, e1000360. 19180233 10.1371/journal.pgen.1000360 PMC2628742

[3] Reiner, A.P., Lettre, G., Nalls, M.A., Ganesh, S.K., Mathias, R., Austin, M.A., Dean, E., Arepalli, S., Britton, A., Chen, Z. et al. (2011) Genome-wide association study of white blood cell count in 16,388 African Americans: the continental origins and genetic epidemiology network (COGENT). PLoS Genet., 7, e1002108. 21738479 10.1371/journal.pgen.1002108 PMC3128101

[4] Charles, B.A., Hsieh, M.M., Adeyemo, A.A., Shriner, D., Ramos, E., Chin, K., Srivastava, K., Zakai, N.A., Cushman, M., McClure, L.A. et al. (2018) Analyses of genome wide association data, cytokines, and gene expression in African-Americans with benign ethnic neutropenia. PLoS One, 13, e0194400. 29596498 10.1371/journal.pone.0194400 PMC5875757

[5] Pierron, D., Heiske, M., Razafindrazaka, H., Pereda-Loth, V., Sanchez, J., Alva, O., Arachiche, A., Boland, A., Olaso, R., Deleuze, J.F. et al. (2018) Strong selection during the last millennium for African ancestry in the admixed population of Madagascar. Nat. Commun., 9, 932. 29500350 10.1038/s41467-018-03342-5 PMC5834599

[6] Legge, S.E., Pardinas, A.F., Helthuis, M., Jansen, J.A., Jollie, K., Knapper, S., MacCabe, J.H., Rujescu, D., Collier, D.A., O'Donovan, M.C. et al. (2019) A genome-wide association study in individuals of African ancestry reveals the importance of the Duffy-null genotype in the assessment of clozapine-related neutropenia. Mol. Psychiatry, 24, 328–337. 30647433 10.1038/s41380-018-0335-7

[7] Duchene, J., Novitzky-Basso, I., Thiriot, A., Casanova-Acebes, M., Bianchini, M., Etheridge, S.L., Hub, E., Nitz, K., Artinger, K., Eller, K. et al. (2017) Atypical chemokine receptor 1 on nucleated erythroid cells regulates hematopoiesis. Nat. Immunol., 18, 753–761. 28553950 10.1038/ni.3763 PMC5480598

[8] Permanyer, M., Bosnjak, B. and Forster, R. (2018) Dual role for atypical chemokine receptor 1 in myeloid cell hematopoiesis and distribution. Cell. Mol. Immunol., 15, 399–401. 29532789 10.1038/cmi.2017.79 PMC6052837

[9] Ortiz, M.V., Meier, E.R. and Hsieh, M.M. (2016) Identification and clinical characterization of children with benign ethnic neutropenia. J. Pediatr. Hematol. Oncol., 38, e140–e143. 26925714 10.1097/MPH.0000000000000528 PMC5102334

[10] Weingarten, M.A., Kahan, E. and Brauner, A. (1992) Normal fluctuations of leucocyte counts and the response to infection in benign familial leucopenia. Acta Haematol., 87, 126–128. 1642093 10.1159/000204738

[11] Shoenfeld, Y., Ben-Tal, O., Berliner, S. and Pinkhas, J. (1985) The outcome of bacterial infection in subjects with benign familial leukopenia (BFL). Biomed. Pharmacother., 39, 23–26. 4027348

[12] Palmblad, J. and Hoglund, P. (2018) Ethnic benign neutropenia: a phenomenon finds an explanation. Pediatr. Blood Cancer, 65, e27361. 30117263 10.1002/pbc.27361

[13] Orsi, J.M., Margellos-Anast, H. and Whitman, S. (2010) Black-White health disparities in the United States and Chicago: a 15-year progress analysis. Am. J. Public Health, 100, 349–356. 20019299 10.2105/AJPH.2009.165407 PMC2804622

[14] Hershman, D., McBride, R., Jacobson, J.S., Lamerato, L., Roberts, K., Grann, V.R. and Neugut, A.I. (2005) Racial disparities in treatment and survival among women with early-stage breast cancer. J. Clin. Oncol., 23, 6639–6646. 16170171 10.1200/JCO.2005.12.633

[15] Kelly, D.L., Kreyenbuhl, J., Dixon, L., Love, R.C., Medoff, D. and Conley, R.R. (2007) Clozapine underutilization and discontinuation in African Americans due to leucopenia. Schizophr. Bull., 33, 1221–1224. 17170061 10.1093/schbul/sbl068 PMC2632351

[16] Rappoport, N., Simon, A.J., Amariglio, N. and Rechavi, G. (2019) The Duffy antigen receptor for chemokines, ACKR1,- 'Jeanne DARC' of benign neutropenia. Br. J. Haematol., 184, 497–507. 30592023 10.1111/bjh.15730

[17] Sudlow, C., Gallacher, J., Allen, N., Beral, V., Burton, P., Danesh, J., Downey, P., Elliott, P., Green, J., Landray, M. et al. (2015) UK biobank: an open access resource for identifying the causes of a wide range of complex diseases of middle and old age. PLoS Med., 12, e1001779. 25826379 10.1371/journal.pmed.1001779 PMC4380465

[18] Pedersen, C.B., Bybjerg-Grauholm, J., Pedersen, M.G., Grove, J., Agerbo, E., Baekvad-Hansen, M., Poulsen, J.B., Hansen, C.S., McGrath, J.J., Als, T.D. et al. (2018) The iPSYCH2012 case-cohort sample: new directions for unravelling genetic and environmental architectures of severe mental disorders. Mol. Psychiatry, 23, 6–14. 28924187 10.1038/mp.2017.196 PMC5754466

[19] Manu, P., Sarvaiya, N., Rogozea, L.M., Kane, J.M. and Correll, C.U. (2016) Benign ethnic neutropenia and clozapine use: a systematic review of the evidence and treatment recommendations. J. Clin. Psychiatry., 77, e909–e916. 27464332 10.4088/JCP.15r10085

[20] DeSantis, C.E., Miller, K.D., Sauer, A.G., Jemal, A. and Siegel, R.L. (2019) Cancer statistics for African Americans, 2019. CA Cancer J. Clin., 69, 211–233. 30762872 10.3322/caac.21555

[21] Wicinski, M. and Weclewicz, M.M. (2018) Clozapine-induced agranulocytosis/granulocytopenia: mechanisms and monitoring. Curr. Opin. Hematol., 25, 22–28. 28984748 10.1097/MOH.0000000000000391

[22] Fry, A., Littlejohns, T.J., Sudlow, C., Doherty, N., Adamska, L., Sprosen, T., Collins, R. and Allen, N.E. (2017) Comparison of sociodemographic and health-related characteristics of UK biobank participants with those of the general population. Am. J. Epidemiol., 186, 1026–1034. 28641372 10.1093/aje/kwx246 PMC5860371

[23] Pedersen, C.B. (2011) The Danish civil registration system. Scand. J. Public. Health., 39, 22–25. 21775345 10.1177/1403494810387965

[24] McCarthy, S., Das, S., Kretzschmar, W., Delaneau, O., Wood, A.R., Teumer, A., Kang, H.M., Fuchsberger, C., Danecek, P., Sharp, K. et al. (2016) A reference panel of 64,976 haplotypes for genotype imputation. Nat. Genet., 48, 1279–1283. 27548312 10.1038/ng.3643 PMC5388176

[25] Bycroft, C., Freeman, C., Petkova, D., Band, G., Elliott, L.T., Sharp, K., Motyer, A., Vukcevic, D., Delaneau, O., O'Connell, J. *et al.* (2017) Genome-wide genetic data on ~500,000 UK biobank participants. bioRxiv, in press.

[26] Howie, B., Fuchsberger, C., Stephens, M., Marchini, J. and Abecasis, G.R. (2012) Fast and accurate genotype imputation in genome-wide association studies through pre-phasing. Nat. Genet., 44, 955–959. 22820512 10.1038/ng.2354 PMC3696580

[27] Genomes Project Consortium, Auton, A., Brooks, L.D., Durbin, R.M., Garrison, E.P., Kang, H.M., Korbel, J.O., Marchini, J.L., McCarthy, S., McVean, G.A. et al. (2015) A global reference for human genetic variation. Nature, 526, 68–74. 26432245 10.1038/nature15393 PMC4750478

[28] Lynge, E., Sandegaard, J.L. and Rebolj, M. (2011) The Danish National Patient Register. Scand. J. Public. Health., 39, 30–33. 21775347 10.1177/1403494811401482

