# Supplementary material for: The Duffy-null genotype and risk of infection
Source: Hum Mol Genet. 2020 Sep 22;29(20):3341–9. doi: 10.1093/hmg/ddaa208 (PMC7906776; doi:10.1093/hmg/ddaa208)
Supplement: HMG_Duffy-null_and_Infection_Supplementary_Materials_REVISED_ddaa208 [file hmg_duffy-null_and_infection_supplementary_materials_revised_ddaa208.docx]

**Supplementary Material**

The Duffy-null Genotype and Risk of Infection

Supplementary Results 2

Secondary analyses including all ethnicities 2

Supplementary Figures 3

Supplementary Figure 1: UK Biobank genetic principal components 3

Supplementary Figure 2: iPSYCH year of birth 4

Supplementary Tables 5

Supplementary Table 1: UK Biobank self-reported ethnicity 5

Supplementary Table 2: UK Biobank country of birth 6

Supplementary Table 3: iPSYCH parental origin 7

Supplementary Table 4: iPSYCH year of birth 8

Supplementary Table 5: UK Biobank infections in all ethnicities 9

Supplementary Table 6: Risk of viral infection in Duffy-null carriers with low neutrophil counts 10

Supplementary Table 7: Risk of infection by neutrophil count 11

Supplementary Table 8: List of countries at current or historical risk of malaria in the Danish Civil Registration System 12

Supplementary Table 9: ICD-10 infection codes 13

## Supplementary Results

### Secondary analyses including all ethnicities

Primary analyses using the UK Biobank sample included individuals with a self-reported Black African/Caribbean ethnicity. However, we also repeated analyses including individuals from all ethnicities to ensure generalisability of the findings.

#### Duffy-null genotype and neutrophil counts

The Duffy-null genotype was highly predictive of ANC in UK Biobank individuals of all ethnicities (β = -1.67; 95% CI = -1.75,-1.60; P < 1 x 10^-300^). Mean ANC in individuals with the CC and TC/TT genotype was 2.81 (standard deviation (SD) = 1.02) and 4.22 (SD = 1.34), respectively. Individuals of all ethnicities with the CC genotype were significantly more likely to have an ANC below 2.0 x 10^9^/L, the lower threshold for a ‘normal’ ANC (OR = 32.44; 95% CI = 22.86,46.04; P = 1.37 x 10^-84^) and 1.5 x 10^9^/L (OR = 45.22; 95% CI = 23.31,87.73; P = 1.78 x 10^-29^). However, individuals with the Duffy-null genotype were not at increased risk for agranulocytosis (ANC < 0.5 x 10^9^/L, OR = 0.44; 95% CI = 0.07,2.98; P = 0.40).

#### Duffy-null genotype and risk of serious infection

In UK Biobank, the CC (Duffy-null) genotype did not increase the risk of a serious infection when including individuals of all ethnicities (rate ratio (RR) = 0.90; 95% CI = 0.80-1.00; P = 0.057; Supplementary Table 5). This was consistent for all types and sites of infection (Supplementary Table 5). We also found no evidence that the Duffy-null genotype increased the total number of serious infections when including individuals of all ethnicities (mean number of infection per individual 0.27 vs. 0.26; RR = 0.91; 95% CI = 0.82-1.02; P = 0.09). Furthermore, individuals with the CC genotype in comparison to the TC/TT genotype in UK Biobank were not significantly more likely to have died from an infection-related illness when including individuals of all ethnicities (0.21% (*n*=16) vs. 0.44% (*n*=2111); RR = 1.04; 95% CI = 0.31-3.55; P = 0.94).

## Supplementary Figures

### Supplementary Figure 1: UK Biobank genetic principal components


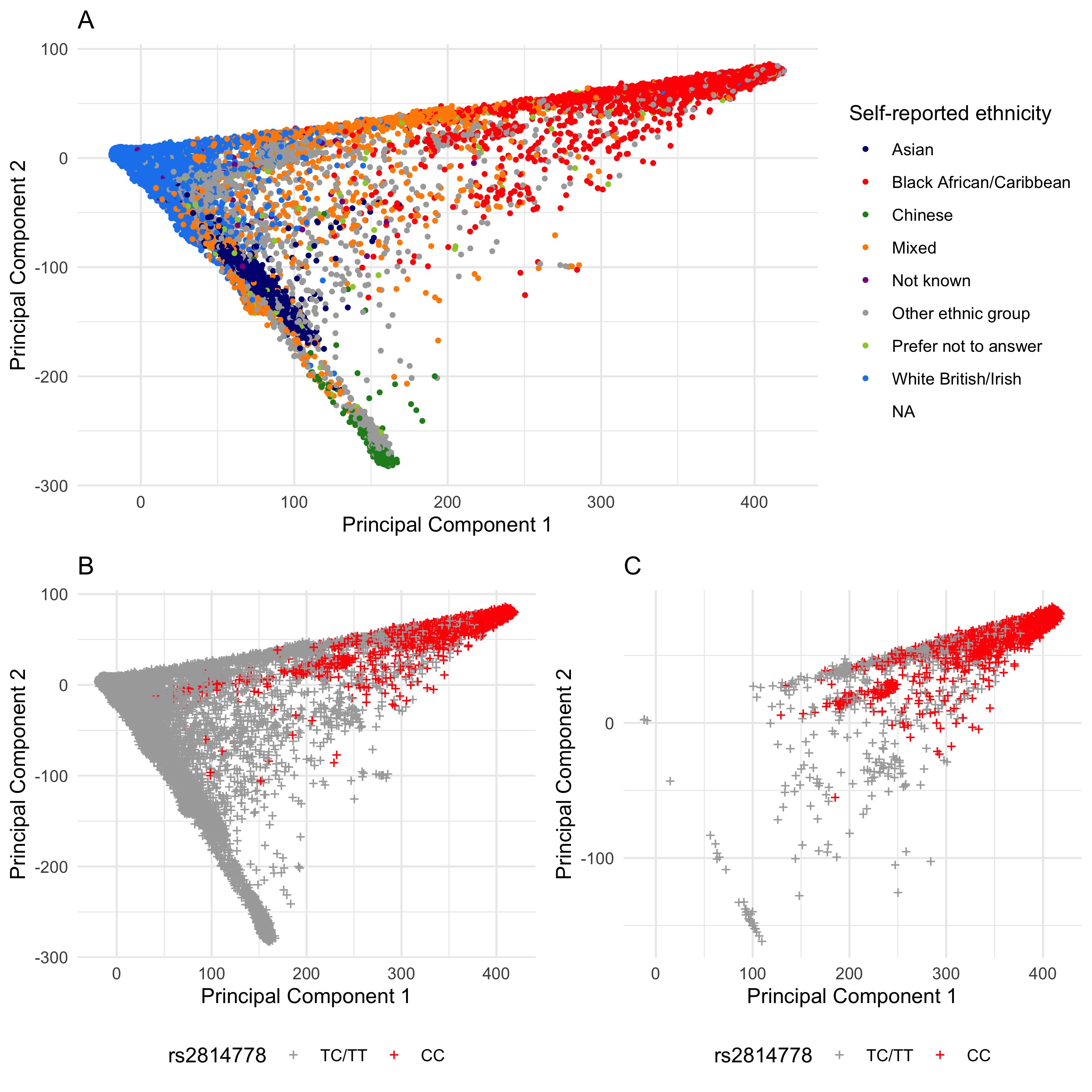


Genetic principal components, self-reported ethnicity and the Duffy-null genotype. Plot A: Principal component 1 vs. principal component 2 for the UK Biobank sample, points coloured by self-reported ethnicity. Plot B: Principal component 1 vs. principal component 2 for the UK Biobank sample, points coloured by rs2814778 genotype (grey = TC/TT, red = CC (Duffy-null)). Plot C: Principal component 1 vs. principal component 2 for UK Biobank individuals who reported a Black African/Caribbean ethnicity, points coloured by rs2814778 genotype (grey = TC/TT, red = CC (Duffy-null)).

### Supplementary Figure 2: iPSYCH year of birth

##
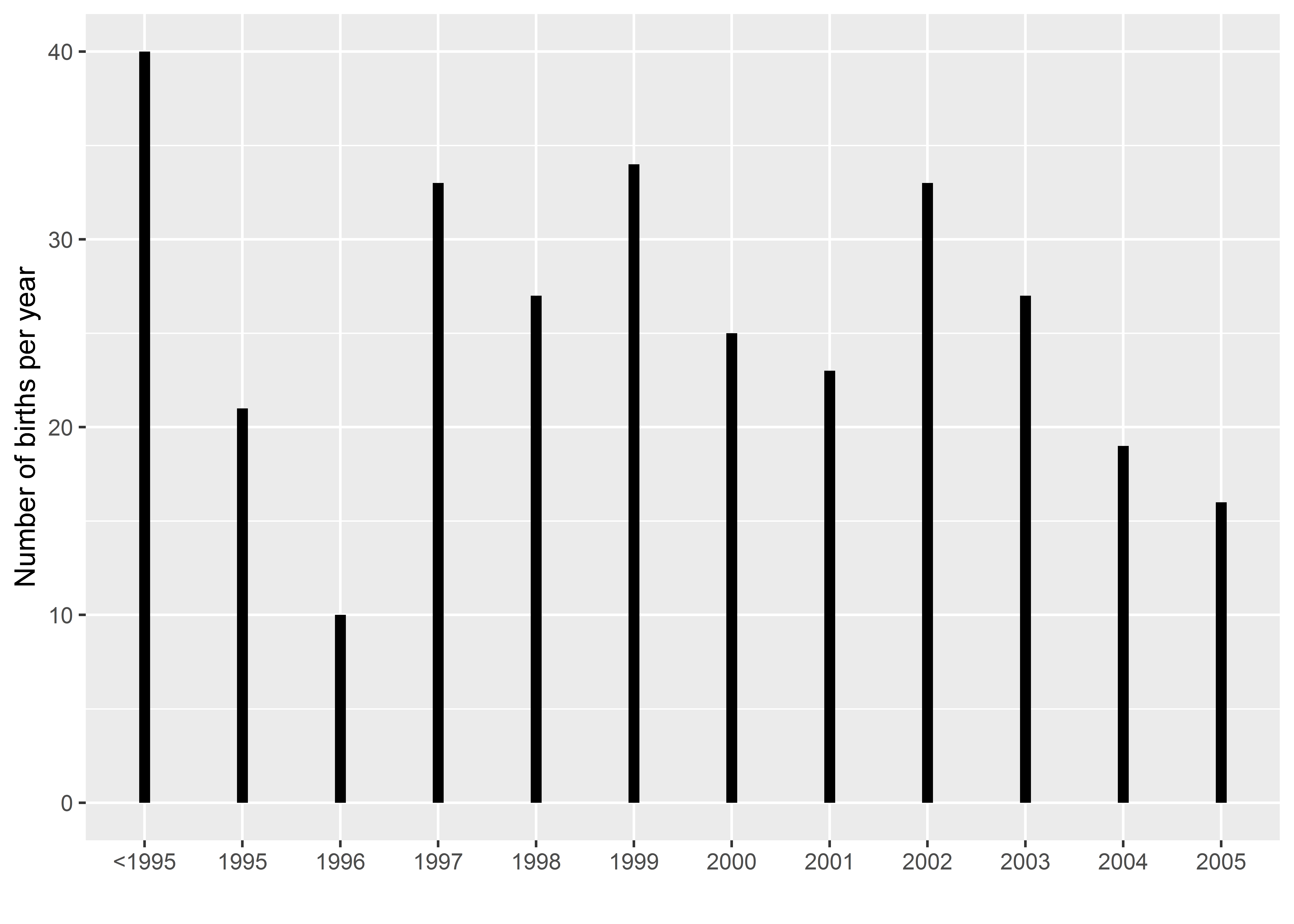


Number of births by year (calendar time) of study individuals in iPSYCH cohort. This is broken down by genotype for rs2814778 in Supplementary Table 5.

## Supplementary Tables

### Supplementary Table 1: UK Biobank self-reported ethnicity

| **Self-reported ethnicity** | **CC (Duffy-null)** | **TC/TT** | **Total** |
| --- | --- | --- | --- |
| White British or Irish | 19 | 459234 | 459253 |
| Black African/Caribbean | 6363 | 1281 | 7644 |
| White and Black African/Caribbean | 36 | 963 | 999 |
| Asian | 0 | 7670 | 7670 |
| Chinese | 0 | 1503 | 1503 |
| Other ethnic group | 800 | 3554 | 4354 |
| Other mixed ethnic group | 26 | 1818 | 1844 |
| Not known | 15 | 189 | 204 |
| Prefer/did not to answer | 139 | 3191 | 3330 |
| **Total** | **7398** | **479403** | **486801** |

Self-reported ethnicity of Duffy-null and non-Duffy-null carriers in UK Biobank.

### Supplementary Table 2: UK Biobank country of birth

| **Country of birth** | **CC (Duffy-null)** | **TC/TT** | **Total** |
| --- | --- | --- | --- |
| North Africa | 3 | 4 | 7 |
| Sub-Saharan Africa | 2728 | 126 | 2854 |
| Caribbean | 1900 | 578 | 2478 |
| UK | 1698 | 553 | 2251 |
| Other | 34 | 20 | 54 |
| **Total** | **6363** | **1281** | **7644** |

Country of birth for UK Biobank individuals with Black African/Caribbean ethnicity, divided into those with the CC and TC/TT genotype for rs2814778. Data extracted from UK Biobank field IDs 20115 (non-UK origin) and 1647 (UK origin).

### Supplementary Table 3: iPSYCH parental origin

| **Parental origin** | **CC (Duffy-null)** | **TC/TT** | **Total** |
| --- | --- | --- | --- |
| *Ancestry region* |  |  |  |
| 2 African parents | 228 | 201 | 429 |
| 2 Middle Eastern parents | 27 | 688 | 715 |
| 0/1 African or Middle Eastern parents | 28 | 76282 | 76310 |
| Total | 283 | 77171 | 77454 |
|  |  |  |  |
| *African region* |  |  |  |
| **2 Malaria belt parents** | **217** | **64** | **281** |
| 1 Malaria belt parent | 21 | 285 | 306 |
| Parents from other regions | 45 | 76822 | 76867 |
| Total | 283 | 77171 | 77454 |

Parental origin for individuals in the iPSYCH sample

### Supplementary Table 4: iPSYCH year of birth

| **Year of birth** | **CC (Duffy-null)** | **TC/TT** | **Total** |
| --- | --- | --- | --- |
| 1981-1995 | 39 | 19 | 58 |
| 1995-1998 | 52 | 7 | 59 |
| 1998-2001 | 58 | 19 | 77 |
| 2001-2005 | 68 | 19 | 87 |
| Total | 217 | 64 | 281 |

Year of birth of study individuals in iPSYCH case-cohort sample.

### Supplementary Table 5: UK Biobank infections in all ethnicities

|  | CC (n=7450)  N (%) | TC/TT (n=479873)  N (%) | RR (95% CI) | SE | P |
| --- | --- | --- | --- | --- | --- |
| Any infection | 1408 (18.90%) | 95870 (19.98%) | 0.90 (0.79-1.02) | 0.06 | 0.09 |
| *Type of infection* |  |  |  |  |  |
| Bacterial | 695 (9.33%) | 41384 (8.62%) | 0.89 (0.74-1.07) | 0.09 | 0.22 |
| Viral | 331 (4.44%) | 17874 (3.72%) | 1.03 (0.78-1.36) | 0.14 | 0.81 |
| Other | 437 (5.87%) | 30878 (6.43%) | 0.98 (0.77-1.24) | 0.12 | 0.87 |
| *Site of infection* |  |  |  |  |  |
| Respiratory | 459 (6.16%) | 38904 (8.11%) | 0.92 (0.74-1.15) | 0.11 | 0.46 |
| Skin | 269 (3.61%) | 21553 (4.49%) | 0.84 (0.63-1.12) | 0.15 | 0.23 |
| Gastrointestinal | 250 (3.36%) | 17802 (3.71%) | 0.96 (0.71-1.30) | 0.16 | 0.78 |
| Sepsis | 76 (1.02%) | 5193 (1.08%) | 1.11 (0.61-2.01) | 0.30 | 0.73 |
| Hepatitis | 72 (0.97%) | 2491 (0.52%) | 1.04 (0.59-1.85) | 0.29 | 0.88 |

Association between infections and Duffy-null genotype for whole UK Biobank sample (all ethnicities included).

### Supplementary Table 6: Risk of viral infection in Duffy-null carriers with low neutrophil counts

| *Low ANC group* | | | |  | *Comparative group (ANC 2.0 – 7.5 x 10^9^/L)* | | |  |  |  |
| --- | --- | --- | --- | --- | --- | --- | --- | --- | --- | --- |
| Genotype | ANC range (10^9^/L) | N of subjects | N with viral infection (%) |  | Genotype | N of subjects | N with viral infection (%) | RR (95% CI) | SE | P |
| CC | < 1.0 | 39 | 5 (12.81%) |  | CC | 5523 | 223 (4.04%) | 3.05 (1.24-7.52) | 0.46 | 0.015 |
|  |  |  |  |  | TC/TT | 444715 | 16244 (3.65%) | 3.18 (1.27-7.95) | 0.47 | 0.013 |
| CC | 1.0 – 1.5 | 323 | 25 (7.74%) |  | CC | 5523 | 223 (4.04%) | 1.92 (1.27-2.91) | 0.21 | 2.13x10^-3^ |
|  |  |  |  |  | TC/TT | 444715 | 16244 (3.65%) | 1.87 (1.16-3.01) | 0.24 | 0.011 |
| CC | 1.5 – 2.0 | 1115 | 57 (5.11%) |  | CC | 5523 | 223 (4.04%) | 1.29 (0.96-1.73) | 0.15 | 0.090 |
|  |  |  |  |  | TC/TT | 444715 | 16244 (3.65%) | 1.19 (0.82-1.74) | 0.19 | 0.361 |
| TC/TT | < 1.0 | 718 | 43 (5.99%) |  | TC/TT | 444715 | 16244 (3.65%) | 1.65 (1.23-2.23) | 0.15 | 9.54x10^-4^ |
| TC/TT | 1.0 – 1.5 | 990 | 67 (6.77%) |  | TC/TT | 444715 | 16244 (3.65%) | 1.84 (1.45-2.35) | 0.12 | 5.68x10^-7^ |
| TC/TT | 1.5 – 2.0 | 5659 | 233 (4.12%) |  | TC/TT | 444715 | 16244 (3.65%) | 1.11 (0.98-1.27) | 0.07 | 0.102 |

Association of viral infection in individuals with the CC genotype and a low ANC compared to individuals with the CC and TC/TT genotype with a normal ANC (2.0 - 7.5 x 10^9^/L). Columns represent firstly for low ANC groups: genotype at rs2814778, ANC range, number of subjects in said group, and number of infections. The comparative group consisted of individuals with an ANC in the range of 2.0 – 7.5 x 10^9^/L and columns represent: genotype at rs2814778, number of subjects and number of infections. RR = rate ratio, in reference to low ANC group; SE = standard error; P = association p-value.

### Supplementary Table 7: Risk of infection by neutrophil count

|  | CC genotype | | |  | TC/TT genotype | | |  |  |  |  |  |
| --- | --- | --- | --- | --- | --- | --- | --- | --- | --- | --- | --- | --- |
| Neutrophil count (10^9^/L) | Total N | N with infections (%) | N of infections |  | Total N | N with infections | N of infections |  | RR (95% CI) | SE | P | |
| 1.0 – 1.5 | 323 | 71 (21.98%) | 85 |  | 990 | 239 (24.14%) | 397 |  | 0.80 (0.23 – 2.72) | 0.63 | 0.719 | |
| 1.5 – 2.0 | 1115 | 194 (17.40%) | 268 |  | 5659 | 1064 (18.80%) | 1402 |  | 0.91 (0.44 – 1.90) | 0.37 | 0.809 | |
| 2.0 – 3.0 | 2973 | 503 (16.92%) | 688 |  | 68125 | 12264 (18.00%) | 15411 |  | 0.85 (0.63 – 1.15) | 0.15 | 0.290 | |
| 3.0 – 4.0 | 1697 | 333 (19.62%) | 491 |  | 146782 | 27059 (18.43%) | 33595 |  | 0.97 (0.77 – 1.23) | 0.12 | 0.822 | |
| 4.0 – 5.0 | 628 | 145 (23.09%) | 217 |  | 126290 | 24960 (19.76%) | 32065 |  | 1.08 (0.83 – 1.39) | 0.13 | 0.571 | |
| 5.0 – 7.5 | 225 | 58 (25.75%) | 99 |  | 103518 | 23012 (22.23%) | 31528 |  | 1.18 (0.87 – 1.60) | 0.16 | 0.300 | |
| 7.5 – 10.0 | 14 | 4 (28.57%) | 13 |  | 10894 | 3170 (29.10%) | 5038 |  | 1.01 (0.38 – 2.70) | 0.50 | 0.988 | |

Risk of infection in UK Biobank by genotype at rs2814778 and neutrophil count. Individuals of all ethnicities are included due to low numbers of TC/TT carriers of Black African/Caribbean ethnicity. Columns represent neutrophil count, then separately for CC and TC/TT carriers for rs2814778, the total number of individuals, the total number of individuals with a serious infection and the percentage of individuals with an infection.

### Supplementary Table 8: List of countries at current or historical risk of malaria in the Danish Civil Registration System

| **Country** |
| --- |
| Federal Democratic Republic of Ethiopia |
| Federal Republic of Nigeria |
| Republic of Angola |
| Republic of Burundi |
| Republic of Cameroon |
| Republic of Cõte d'Ivoire |
| Republic of Djibouti |
| Republic of Ghana |
| Republic of Kenya |
| Republic of Liberia |
| Republic of Madagascar |
| Republic of Malawi |
| Republic of Mali |
| Republic of Mauritius |
| Republic of Mozambique |
| Republic of Namibia |
| Republic of Rwanda |
| Republic of Senegal |
| Republic of Sierra Leone |
| Republic of the Sudan |
| Republic of Uganda |
| Republic of Zambia |
| Republic of Zimbabwe |
| Somali Republic |
| State of Eritrea |
| The Democratic Republic of Congo |
| The Gambia |
| Togolese Republic |
| Union of the Comoros |
| United Republic of Tanzania |

### Supplementary Table 9: ICD-10 infection codes

| Infection category | ICD-8 Codes | ICD-10 Codes |
| --- | --- | --- |
| **Site of infection** | | |
| Sepsis infections | 038 | A40-A41 |
| Hepatitis infections | 070 | B15-B19, K770 |
| Gastrointestinal infections | 000-009, 540 | A00-A09, K35 |
| Skin infections | 035, 050-057, 110-111, 680-686 | A46, B00-B09, L00-L08 |
| Respiratory infections | 460-486 | J00-J18, J22, J36 |
| Urological infections | 580, 590, 595 | N00, N05, N300, N370, N390, N129 |
| Genital infections | 604, 612, 620, 622 | N45, N51.2, N70, N70.0, N70.9, N76.0, N76.2, N76.4, N77.0, N77.1, N518B |
| Pregnancy-related infections | 630, 635, 670 | O23, O26.4, O85–O86, O98 |
| Otitis media infections | 381-382 | H65-H67 |
| Central nervous system infections | 013, 027.01, 036.09, 040–043, 045-046, 052.01, 053.02, 054.03, 055.01, 056.01, 062-065, 071.99, 072.02, 075.01, 079.29, 090.49, 094.9, 320, 322–324,  474 | A02.2C A06.6, A17, A229C, A32.1, A39.0, A50.4, A514B, A521A-B, A521B, A548A, A548D, A80-89, B00.3-B00.4, B01.0- B01.1, B02.0- B02.1, B05.0-B05.1, B06.0, B26.1-B26.2, B37.5, B45.1, B58.2, B60.2, E236A, G00-G07 |
| **Type of infection** | | |
| Bacterial infections | 000-005, 008.09-008.39, 010-018, 020-023, 025-039, 073.99, 076.99, 079.84, 080-083, 088.99-089, 090.09-090.59, 091-098, 100-104, 320.09-320.80, 322, 380-382, 390-392, 420-421, 461- 464.03, 481- 483, 501.99, 508.00-508.03, 510, 513, 522.59, 526.49, 527.22, 528.38-528.39, 529.03, 540.01, 566-577.03, 590, 595.00-595.01, 597.00, 597.03, 599, 601, 604, 607.39, 611.00-611.01, 612, 614, , 620.90, 620.99, 622, 629.49, 630-631.39, 635, 670,678.01, 680, 681.08-683, 684.08-684.09, 685.01, 686.00-686.08, 710, 720.00-720.29, 720.31, 732 | A022C, A03-A05, A15-A58, A65-A79, B088D, B95-B96 , D733, E060A, E236A E321, , I301A-D, I320, I398, I410, I430, I520A, J01, J020, J030, J13-J15, J160, J170, J851, J86, K61, K67, , K040A, K046A, K052A, , K112A, K113, K122, K130A, K140A, K209A, K351, K650N, K630, K930, L00-L04, L08, M00, M010-M013, M015B, , M463, M490-M492, M680, M725A, N10-N12, N136, N151, N200I, N201I, N300, N341, N390, N410, N412, N431, N450, N459, N510A,C, N511, N70-N74, N764, N980, O23, O753, O85-O86, O980-O982, T814A-D, T814F-J, T793, T802, T874, T880 |
| Viral infections | 008.80, 008.89, 008.90, 040-046, 050-065, 067- 072, 074-075, 078, 079.82, 460,464, 465, 470-480 | A08, A60, A630, A80-A89, A90-A99, B00, B01-B09, B15-B27, B33-B34, B97, G020, G051, H621 A-B, H671 A-B, I400B, I411 A-B, J00, J04-J06, J10-J12, J171, J203-J207, J210, K770A-B, K871A-B, M014-M015, N518B, N770D, N771B, N771G, N771L |
| Other infections | 006-007, 008.99, 009.99, 084, 087, 089, 099.91-099.99, 110-117-131, 136.00, 136.03, 572, 998.59 | A06-A07, A085, A09, A59, A63-A64, B35-60, B64-B83, B87-B89, B99, G02, G040, G049, G052A-C, G052E -J, G079D, H622, I301, I33, I400, I411-412, I521C, J02-J03, J18, J172, J173, J178, J22, J998B-C, K35, K770C-E, K750, L303, M016, M631C-F, M632A, M651, M711, N160D, N370A, O983, O986-O989, T89 |

ICD codes used to extract infection. The same codes were used for both UK Biobank and iPSYCH samples.
